# PROTOCOL: Occupational health and safety regulatory interventions to improve the work environment: An evidence and gap map of effectiveness studies

**DOI:** 10.1002/cl2.1231

**Published:** 2022-04-12

**Authors:** Anja Bondebjerg, Trine Filges, Jan H. Pejtersen, Bjørn C. A. Viinholt, Hermann Burr, Peter Hasle, Emile Tompa, Kirsten Birkefoss, Elizabeth Bengtsen

**Affiliations:** ^1^ VIVE—The Danish Center for Social Science Research Copenhagen Denmark; ^2^ Federal Institute for Occupational Safety and Health (BAuA) Berlin Germany; ^3^ University of Southern Denmark Odense Denmark; ^4^ Institute for Work and Health Toronto Canada

## Abstract

This is the protocol for a Campbell systematic review. The objectives are as follows: provide an overview of the existing evidence base by identifying available systematic reviews and primary effectiveness studies, identify clusters of evidence suitable for a systematic review and identify gaps in evidence where primary research is needed.

## 1 INTRODUCTION

### 1.1 The problem, condition or issue

Contraventions of occupational health and safety regulatory standards pose significant regulatory challenges to lawmakers and working environment authorities on a global scale. Around the world, unsafe working conditions, including, for example, exposure to hazardous materials, use of dangerous equipment, and adverse psychosocial working environments, lead to both human and financial losses that put a strain on labour markets and public welfare institutions. The International Labour Organisation (ILO) estimates that 2.78 million people die each year due to occupational accidents or work‐related diseases, with an additional yearly 374 million nonfatal work‐related injuries (The International Labour Organisation [ILO], 2020b). While the severe human costs are self‐evident, the economic price of flawed occupational health and safety practices is at approximately 3.94% of global GDP each year according to the ILO (2020b).

With the adverse effects of contraventions of occupational health and safety regulatory standards being indisputable, there is a need to ensure that research about what constitutes effective occupational health and safety regulatory interventions is available to policymakers, regulatory agencies, and other key stakeholders. Several studies have reviewed the literature on the effects of regulatory enforcement tools such as workplace inspections, consultative activities, and awareness campaigns. Tompa et al. (2007) performed a systematic review of insurance and regulatory mechanisms, which was followed up by two reviews published in 2016, focusing respectively on the quantitative and the qualitative regulatory enforcement literature (MacEachen et al., 2016; Tompa et al., 2016). Findings from the 2007‐review found evidence that first hand experience of citations and penalties (which can be defined as specific deterrence) reduced injuries, whereas there was limited evidence for the effects of first hand experience of inspection. Similarly, there was limited evidence for the probability of inspections, citations, and penalties (defined as general deterrence) leading to reduced injuries (Tompa et al., 2007). The quantitative review from 2016 also found evidence to suggest that several regulatory enforcement mechanisms had an effect on injuries and compliance with regulation. Particularly, there was a specific deterrence effect from inspections with penalties which resulted in a decrease in injuries, similar to findings from the 2007‐review (Tompa et al., 2016). Results from a Cochrane intervention review by Mischke et al. (2013) were also in line with the findings from the above mentioned reviews, in that evidence was found for inspections leading to decreases in injuries, with indications of specific, focused inspections having potentially larger effects than inspections in general. However, this review also pointed to a lack of high‐quality evidence and an urgent need for better designed evaluations to establish the effects of enforcement methods (Mischke et al., 2013). Finally, a systematic review by Andersen et al. (2019) indicated that legislative and regulatory policy may reduce injuries and fatalities and improve compliance with regulation, but major research gaps were identified concerning the effects of occupational health and safety regulation targeting psychological and musculoskeletal disorders.

As can be seen from the above presentation of previous reviews, there is evidence to suggest that regulatory interventions may serve as effective tools for governments tasked with protecting workers from health and safety risks at workplaces. At the same time, it is clear that there are knowledge gaps in the literature which make it hard to determine relationships of cause and effect pertaining to particular intervention and outcome domains. In addition, there is a multitude of different regulatory interventions which may lead to diverse effects on a large number of outcomes, ranging from intermediate outcomes on the organisational level (such as compliance with regulations and workplace exposures) to final outcomes for individual workers (such as the prevalence of ill‐health and injuries). Finally, occupational health and safety regulation as a research field is characterised by methodological diversity, with researchers drawing on various types of both quantitative and qualitative methods to gain insight into the complexities of regulatory interventions. Taken together, the existence of knowledge gaps and the extent and diversity of the research literature can make it challenging for both government stakeholders, regulatory agencies, and researchers alike to gain an overview of what evidence is available for particular interventions. This points to the need for applying methods of knowledge mapping that are suited to the task of creating overviews of large amounts of data in a format that is accessible to various stakeholders. One such method is the evidence and gap map (EGM) approach which we will apply in this protocol.

As stated by Saran and White in their overview of approaches to evidence mapping, evidence maps constitute a relatively new approach to systematically reporting research activity for broader topic areas than those made possible by traditional, focused research syntheses, such as systematic reviews (Saran & White, 2018). Evidence maps are well suited for guiding stakeholders to high‐quality research, identifying research gaps, and pointing out the direction for more focused research syntheses (Saran & White, 2018). The current EGM intends to do just that: to provide an easily accessible, visual representation of the availability of evidence for the effects of a number of occupational health and safety regulatory interventions on a predefined set of workplace and worker level outcomes. In this project, we adopt a functional definition of regulation in which regulation involves: (a) setting regulatory standards, (b) monitoring compliance through inspections, and (c) enforcing regulatory standards (incentives and sanctions). This functional definition is reflected in our intervention framework which covers these three regulatory functions, as well as information and training initiatives initiated by regulatory agencies to improve compliance with regulatory standards. As mentioned, enforcement of working environment regulation can influence a host of outcomes, including both intermediate workplace outcomes and individual worker outcomes. Therefore, this EGM includes a number of outcome domains intended to cover both the intermediate and final outcome levels.

As noted, research in occupational safety and health regulation is characterised by methodological diversity, with strong traditions of both quantitative and qualitative enquiry. However, we will not attempt to cover the occupational health and safety regulatory enforcement literature in its entirety. Our aim with this EGM lies specifically in finding studies designed to make causal inferences about intervention effects. Therefore, we limit our scope to a subset of studies within the larger occupational health and safety regulation literature, that is, primary studies of effectiveness and systematic reviews of effects. This choice has been guided by the input we have received from our stakeholders within The Danish Working Environment Authority, who have expressed a need for a better overview of existing research and a clearer picture of potential research gaps and avenues for future research. If a sufficient number of primary effectiveness studies are located for one or more intervention/outcome areas in this EGM, a possible next step will be to perform a full systematic review and meta‐analysis. As noted by Snilstveit et al. (2016), identifying areas or ‘synthesis gaps’ where systematic reviews can be of particular value are one of the key potentials of EGMs and a fruitful avenue for making research evidence available and useful to stakeholders.

### 1.2 The intervention

As noted, the intervention framework reflects the proposed definition of regulation as involving three main functions: setting regulatory standards, monitoring compliance through inspections, and enforcing regulatory standards (incentives and sanctions). In addition to this, we are interested in other efforts made by regulatory agencies to improve compliance or deter noncompliance, specifically information and training initiatives. In accordance with this, we focus on six types of occupational health and safety regulatory initiatives: (1) formulation of regulatory standards, (2) incentives for compliance, (3) inspection by regulatory agencies, (4) enforcement by regulatory agencies (sanctions), (5) information, guidance, and consulting, and finally (6) training initiatives. We provide further details on the intervention categories in Section 3.7. Our outcomes of interest include both workplace and worker level categories, with examples being compliance with occupational health and safety regulatory standards, exposure to harmful substances, and incidence of work‐related injuries and ill‐health.

### 1.3 Why it is important to develop the EGM

EGMs are a valuable tool in helping researchers and decision makers make sense of the evidence available, supporting the creation of evidence‐informed policies and guiding research prioritisation. We intend this EGM to be of use to both researchers, decision‐makers, and practitioners working in the field of occupational health and safety regulation. To ensure that our work is relevant to stakeholders, we will take guidance from key persons within The Danish Working Environment Authority. Furthermore, we include a group of international researchers with content knowledge expertise as co‐authors of the EGM.

To our knowledge, no EGMs exist that assess the available evidence on the effects of occupational health and safety regulatory interventions. The current EGM will therefore be instrumental in a number of ways, including (1) guiding decision‐makers and other stakeholders to areas covered by sufficient evidence, (2) identifying areas with enough studies to merit a systematic review and meta‐analysis, and finally (3) enabling targeted commissioning of new research in areas characterised by knowledge gaps (Snilstveit et al., 2016).

## 2 OBJECTIVES

The proposed EGM will present available systematic reviews and primary studies examining the effectiveness of interventions aimed at the setting/introduction, monitoring, and/or compliance with occupational health and safety regulations. The three overarching objectives of the map are to:

1. Provide an overview of the existing evidence base by identifying available systematic reviews and primary effectiveness studies,
2. Identify clusters of evidence suitable for a systematic review,
3. Identify gaps in evidence where primary research is needed.

## 3 METHODS

### 3.1 EGM: Definition and purpose

This EGM will provide a visual overview of the available evidence on the effects of a chosen set of occupational health and safety regulatory interventions. In this sense, the EGM will identify what knowledge is available about these interventions by mapping available systematic reviews and primary studies of effectiveness and placing them in a graphical matrix displaying areas characterised by strong, weak, or nonexistent evidence. It is important to note here that an EGM shows the availability of data, but does not synthesise the data. As expressed by Saran and White (2018) in their paper on approaches to EGMs: ‘Evidence maps summarize what evidence there is, not what the evidence says’ (Saran & White, 2018, p. 11).

The present EGM will consist of two primary dimensions: rows listing interventions, and columns listing outcomes, meaning that each cell will show studies containing evidence on that particular combination of intervention and outcome. The map will also contain relevant filters, such as study design or country, making it possible to focus on a subset of studies meeting certain criteria. The map will be populated based on:

- Criteria for inclusion and exclusion of studies.
- Types of studies to be included.
- Quality appraisals of systematic reviews using AMSTAR‐2 (Assessing Methodological Quality of Systematic Reviews; Shea et al., 2017).^1^

### 3.2 Framework development and scope

We will follow the standard EGM framework as a matrix, with rows containing intervention domains and columns containing outcome categories. Both interventions and outcomes will be supplied with subcategories when relevant. Our framework of specific interventions and outcomes, as described in the following, has been developed in cooperation with stakeholders from The Danish Working Environment Authority. It has been our goal to build a framework of relevance, not only to other researchers, but also to decision makers and practitioners who are tasked with developing regulatory standards, performing inspections and enforcing compliance, and taking other measures to improve working environment conditions.

### 3.3 Stakeholder engagement

Engaging primary stakeholders in an EGM can play a central role in defining the scope of the investigation, developing a coherent framework, and making sure that the questions asked are the most relevant and pertinent to the research subject. As noted by Keown et al. (2008), a systematic review (or in this case, an EGM) provides a number of engagement opportunities in various phases of the review process, from cooperating with stakeholders on finding a topic of mutual relevance, to arranging on‐going feedback meetings, and involving stakeholders in the dissemination of the review or EGM to reach, for example, practitioner groups. In the current project, we have drawn on the support of a group of working environment experts within The Danish Working Environment Authority, as they are uniquely positioned to judge what the most pertinent topics are when it comes to enforcing occupational safety and health regulations. In line with Keown et al. (2008), it has been our aim to engage our stakeholders in all parts of the EGM process, starting by consulting with them in the initial process of setting the scope of the EGM and defining relevant outcomes and interventions. This process was initiated in the Fall of 2019 when an open meeting was held between the research team from VIVE and working environment experts from The Danish Ministry of Employment and The Danish Working Environment Authority who expressed their needs for a better overview of existing occupational health and safety research. Having guided us in determining the scope of the EGM, the stakeholders further provided feedback on the title registration and subsequent protocol drafts. This cooperative process has been instrumental in increasing the relevance and precision of the EGM framework. Going forward with the EGM, we will keep our stakeholders updated on the mapping process and the ensuing results, asking for their assessments of, for example, the clarity of presentation and user‐friendliness of the final map. Finally, we will enlist the support of our stakeholders in determining how best to disseminate the results of the investigation to key interest groups.

In addition to the VIVE research team, three international researchers in working environment issues contribute as co‐authors of the EGM:

- Professor Peter Hasle, University of Southern Denmark,
- Senior Scientist Hermann Burr, Federal Institute for Occupational Safety and Health (BAuA), Berlin, Germany,
- Senior Scientist Émile Tompa, Institute for Work & Health, Toronto, Canada.

It is important to state that the involvement of The Danish Ministry of Employment and The Danish Working Environment Authority in this EGM amounts only to consultative support and does not entail ownership or financial support. Any research decisions made are the responsibility of the review authors (i.e., the research team from VIVE and the three international researchers) and the conclusions of this EGM are the sole responsibility of the authors.

### 3.4 Conceptual framework

**Figure 1:**
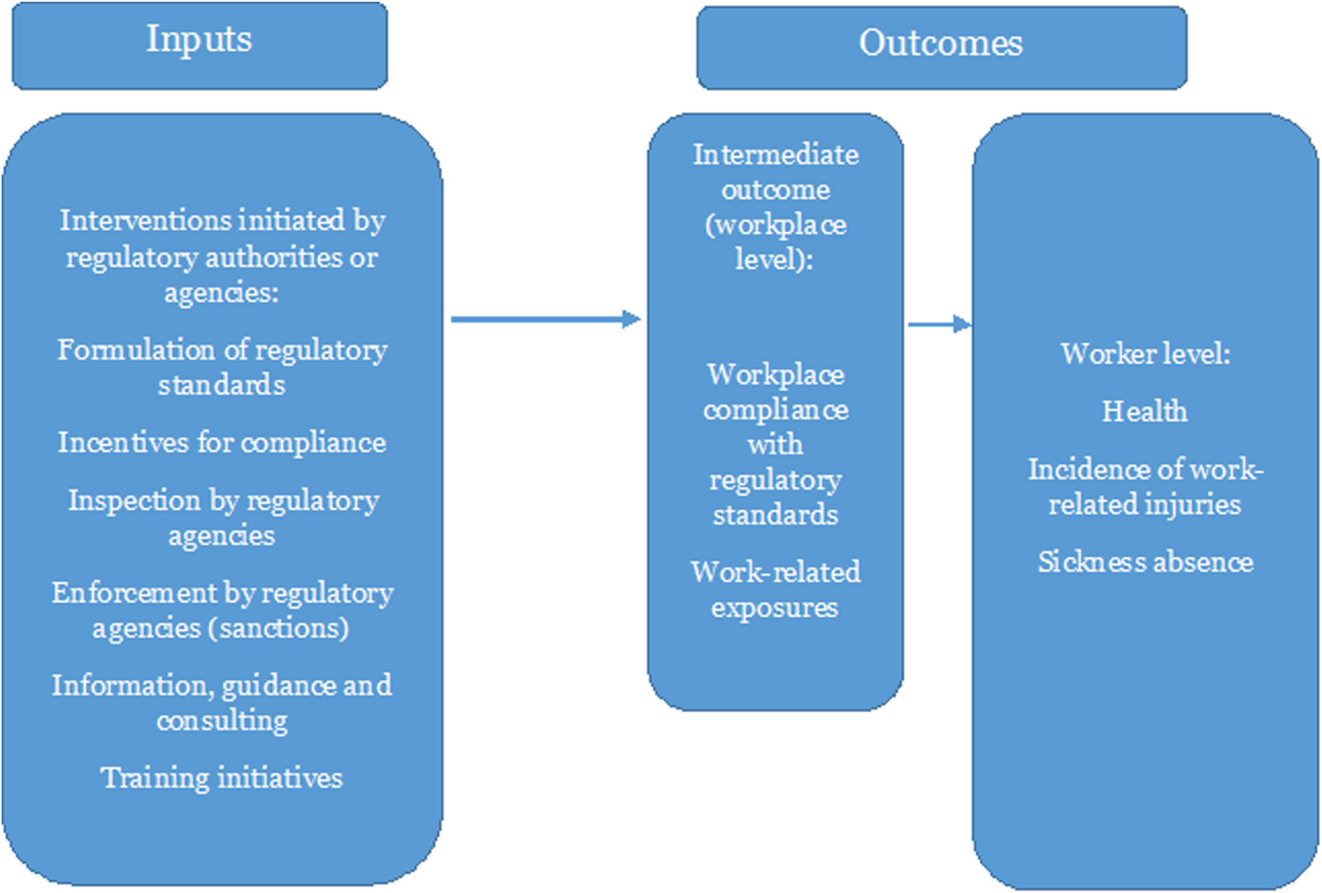
Logic model of interventions and outcomes

Figure 1 shows the logic model for the interventions and how they link to the outcomes. This does not provide a detailed theory of change of how the interventions may work, but provides a conceptual framework of how we imagine that the inputs in focus may lead to the chosen outcomes. As noted previously, we focus on six types of occupational health and safety regulatory initiatives: (1) formulation of regulatory standards, (2) incentives for compliance, (3) inspection by regulatory agencies, (4) enforcement by regulatory agencies (sanctions), (5) information, guidance, and consulting, and finally (6) training initiatives. These six types of initiatives may have an effect on outcomes through processes of general deterrence, which would be the case if, for example, inspections led to an increased general awareness among businesses of the risk of inspection and potential punishment, resulting in increased compliance with regulatory standards. Interventions may also have specific deterrence effects, as would be the case if actual inspections and consequent punishments of noncompliant businesses lead to decreases in violations among those punished (Mischke et al., 2013). As the focus of this EGM is on initiatives by working environment regulatory authorities or regulatory agencies and other organisations assigned as regulators, we will exclude interventions started by individual businesses or employers at their own initiative.

In terms of outcomes, regulatory enforcement is thought to influence health and safety at workplaces both on an organisational (workplace) level and on an individual (worker) level. Workplace compliance with regulatory standards and work‐related exposures are positioned as intermediate outcomes, since the ultimate goal of regulatory enforcement is to reduce negative final outcomes (which are work‐related injuries, health, and sickness absence in this EGM); compliance and reductions in exposure to hazardous materials are essentially means to that end (Tompa et al., 2016). As we work on the map, we will update and make changes to the framework if needed.

### 3.5 Dimensions

As stated previously, this EGM will have two primary dimensions: interventions (rows) and outcomes (columns). Additional dimensions will be:

1. Study designs.
2. Country/region and population subgroups, if relevant.
3. Publication type (report, journal article, etc.).

In the following, we will specify the criteria for determining eligibility of references based on study design, interventions, population, and outcomes.

### 3.6 Types of study design

This EGM focuses on the effectiveness of occupational health and safety regulatory interventions, including measures to ensure compliance and/or deter noncompliance with regulatory standards. To provide an overview of what is known about the effects of such interventions, at the individual and workplace level, we will include all study designs that use a well‐defined control group. We anticipate that relatively few randomised controlled trials on the effects of occupational health and safety regulatory interventions will be found, but such studies will of course be included. The study designs eligible for inclusion in this EGM are:

1. Randomised controlled trials (RCTs).
2. Non‐randomised studies (NRS), where there is a comparison of two or more groups of participants.
3. Systematic reviews (SR) of effects, provided that they report replicable methods to synthesise and summarise the available research evidence and provided that the individual studies fulfil criteria (1) or (2). We will include both systematic reviews in which meta‐analyses were performed and systematic reviews where such analyses were planned, but ended up not being feasible (e.g., due to a low number of included studies). We will exclude systematic reviews in which the authors draw solely on narrative synthesis methods.

We define a systematic review and a meta‐analysis in line with the definitions used by PRISMA and The Cochrane Collaboration, in which it is stated that: ‘(…) a systematic review is a review of a clearly formulated question that uses systematic and explicit methods to identify, select, and critically appraise relevant research, and to collect and analyse data from the studies that are included in the review (…) Meta‐analysis refers to the use of statistical techniques in a systematic review to integrate the results of included studies’^2^ (see also Higgins & Green, 2011). We will appraise the quality of systematic reviews using the AMSTAR‐2 tool (Shea et al., 2017) (see footnote 1). The quality of RCTs and non‐randomised studies will not be assessed, since the purpose of this EGM is to map the literature, not to make decisions based on individual studies (Welch et al., 2019).

Furthermore, studies using single group pre‐post comparisons and non‐randomised studies using an instrumental variable approach will not be included—see the Supporting Information Appendix C (*Justification of exclusion of studies using an instrumental variable (IV) approach*) for our rationale for excluding studies with these designs. A further requirement to all types of studies (randomised as well as non‐randomised) is that they are able to identify an intervention effect. Studies where, for example, the treatment is given to one single organisation/company/workplace only and the comparison group is another organisation/company/workplace (or more organisations/companies/workplaces for that matter) cannot separate the treatment effect from the organisation/company/workplace effect. There shall be at least two organisations/companies/workplaces in both the treatment and comparison group.

Special caution will be taken concerning studies using regression discontinuity designs (RDD) to estimate the treatment effect. In sharp RDDs, a threshold of a (non‐manipulable) forcing/running variable determines which workplaces receive a treatment and which do not, that is, the design is similar to an RCT in the sense that the random sequence determining treatment assignment can be seen as a running variable (Lee & Lemieux, 2008). In contrast, in ‘fuzzy’ RDDs, being on one side of a threshold only makes it more likely that a workplace ends up in the treatment or control group, and the threshold is used as an instrument to estimate local average treatment effects (LATE) (Alper & Karsh, 2009; Imbens & Lemieux, 2008). That is, fuzzy RDD is a form of IV analysis, which we will exclude due to the comparability issues mentioned earlier. Sharp RDDs will be included.

We will not include qualitative research.

Please note that in implementing the above restrictions on types of study designs, we acknowledge that we limit our focus to a selected field of the occupational health and safety literature on regulatory interventions. We are fully aware of the methodological diversity of this study field and of the value generated by using different types of methodologies to gain insight into the complexities of such interventions. An example of this is the contributions made within the sociology of law, represented by, for example, Keith Hawkins (1989), which have helped to advance the study of occupational health and safety regulation.

Our approach in this EGM specifically targets quantitative effect studies. In conducting the EGM and reporting on its' results, we will be careful in clarifying that this project does not aim to give a full and comprehensive overview of the regulatory intervention literature per se; it covers a select subset of studies characterised by the use of distinct, quantitative methods of effect estimation. Should the EGM uncover a sufficient number of effect studies, it may pave the way for focused systematic review and meta‐analysis of particular types of occupational safety and health regulatory enforcement.

### 3.7 Types of intervention/problem

In this EGM, our focus will be exclusively on interventions initiated by working environment regulatory authorities or regulatory agencies and other organisations authorised as regulators, thus excluding interventions started by individual businesses or employers at their own initiative. In Table 1, we present the included intervention categories and examples of subcategories:

**Table 1:** Intervention categories and subcategories

Formulation of regulatory standards	Introduction of occupational safety and health regulatory standards or legislation.
Incentives for compliance	Incentives introduced by regulatory agencies to facilitate compliance with regulatory standards. This includes financial incentives (positive or negative), such as insurance‐related incentives, funding or subsidy programmes, and tax incentives, as well as nonfinancial incentives, such as recognition schemes (e.g., awards). This also covers introduction of certified occupational health and safety management systems.
Inspection by regulatory agencies	Inspections carried out by regulatory agencies. This covers different types of inspections as well as inspections of varying duration, including targeted inspections, and consultative inspections or inspections based on dialogical approaches.
Enforcement by regulatory agencies (sanctions)	Sanctions imposed due to lack of compliance with regulatory standards. This includes fines, legal prosecution, enforceable undertakings, making violations public (e.g., through a red smiley), and orders to comply.
Information, guidance, and consulting	Information, guidance and consulting activities initiated by regulatory agencies targeting, e.g., specific types of workplaces or particular workplace hazards, such as information on important safety procedures at building sites.
Training initiatives	Initiatives taken by regulatory agencies to train or educate workplaces, workers, managers, and other OHS professionals in e.g. safety management or use of mechanical aids or protective gear.

### 3.8 Types of population (as applicable)

The population of relevance to this EGM includes workers above the age of 15 and their workplaces. Conceivably, significant differences exist between the occupational health issues faced by developing nations as opposed to those seen in high‐income countries, which makes it difficult to create a coherent and comparable framework to cover occupational health interventions on a global scale. Therefore, we choose to limit our scope to workplaces located in nations within the OECD. Note here that it is the workplaces that must be located in OECD countries, whereas workers in these workplaces may be citizens of all countries. If possible, we will consider issues of equity and differences in outcomes related to, for example, country GDP within the OECD area, just as we will pay attention to areas of special interest in terms of working environment hazards (e.g., specifically dangerous working environments or areas characterised by particularly high numbers of days lost to psychological ill health).

### 3.9 Types of outcome measures (as applicable)

Contraventions of occupational health and safety regulatory standards and poor working conditions may result in a number of adverse outcomes of which we include the following intermediate and final outcomes:

- *Workplace compliance with regulatory standards (intermediate outcome)*: Workplace compliance with regional, national, or international working environment regulatory standards concerning, for example, the company's health and safety performance, ergonomics, use of technical machinery, and dangerous chemicals, as monitored by regulatory agencies.
- *Work‐related exposures (intermediate outcome)*: Exposure to substances or hazards that carry the risk of damage to worker health. Workers may be exposed to different types of hazards:

o biological hazards, including viruses, bacteria, insects, animals, and so forth, that cause adverse health effects through contact with, for example, mold, blood and other bodily fluids, harmful plants, sewage, dust, and vermin,
o chemical hazards, which include chemical substances resulting in both health and physical impacts such as skin irritation, respiratory problems, and explosions,
o physical hazards, covering environmental factors, for example, noise, radiation, or extreme temperatures,
o safety hazards, which create unsafe working conditions, for example, due to moving machinery or exposed wires,
o ergonomic hazards, which are a result of physical factors, such as manual handling or repetitive, strenuous work positions,
o psychosocial hazards, including harassment, workplace violence, and adverse psychosocial working conditions which may have damaging effects on both physical and mental health and wellbeing (Martinelli, 2020).

- *Health (final outcome)*: Examples of health issues include musculoskeletal disorders, allergies or asthma, hearing loss, and mental ill‐health. Mental ill‐health may include stress symptoms, depressive mood, and absence of wellbeing.
- *Incidence of work‐related injuries (final outcome)*: Covers all injuries, nonfatal or fatal, resulting from incidents arising out of or in the course of work. This includes traffic accidents happening during working hours.
- *Sickness absence (final outcome)* Covers short‐ and long‐term periods of absence from work, for example, measured in terms of work days lost.

### 3.10 Other eligibility criteria

As previously noted, we will only include workplaces located in OECD nations.

### 3.11 Search methods and sources

Relevant studies will be identified through searches in electronic databases, hand search in journals, grey literature searches, searches in repositories and web resources, citation tracking, and contact to international experts. Searches will be aimed at retrieving both published and on‐going studies.

#### 3.11.1 Electronic databases

The following electronic databases will be searched:

- Academic Search (EBSCO)
- EconLit (EBSCO)
- PsycINFO (EBSCO)
- Socindex (EBSCO)
- CINAHL (EBSCO)
- International Bibliography of the Social Sciences(ProQuest)
- Sociological Abstracts (ProQuest)
- Science Citation Index Expanded (Web Of Science)
- Social Sciences Citation Index (Web Of Science)
- MEDLINE (PubMed)
- ERIC (EBSCO)

We have consulted the list of databases comprised in the article by Kugley et al. (2017).

#### 3.11.2 Description of search string

The search string is based on the PICO(s)‐model and contains three concepts, of which we have developed three corresponding search facets: population, intervention, and study type/methodology. The subject terms in the facets will be chosen according to the options available in each database.

#### 3.11.3 Example of a search string

The search string below is developed to search Socindex through the EBSCO search interface and exemplifies the search facets as they will be searched:

#### 3.11.4 Limitations of the search string

There will be no language or time limits to our searches (except in the case of hand searches in journals, which will be limited to 2015‐present, see next section).

**Table:** 

#	Query
S15	S4 AND S10 AND S14
S14	S11 OR S12 OR S13
S13	DE (‘effect size’ OR ‘Control Groups’ OR ‘Experimental Groups’ OR ‘Experiments’ OR ‘Matched Groups’ OR ‘Quasiexperimental Design’ OR ‘Randomized Controlled Trials’ OR ‘Comparative Testing’)
S12	AB (((control* OR difference* OR matched* OR random* OR reference* OR compare* OR longitudinal OR cohort*) N3 (group* OR trial* OR test* OR study OR studies OR analy*))) OR AB ((intervent* OR experiment* OR impact* OR ‘systematic review’ OR ‘meta analy*’ OR metaanaly* OR meta‐analy* OR ‘gap map’ OR ‘follow‐up stud*’ OR ‘follow up stud*’ OR ‘followup stud*’))
S11	TI (control* OR difference* OR matched* OR random* OR reference* OR compare* OR group* OR trial* OR test* OR intervent* OR experiment* OR impact* OR ‘systematic review’ OR ‘meta analy*’ OR metaanaly* OR meta‐analy* OR ‘gap map’ OR study OR studies OR analy* OR longitudinal OR ‘follow‐up stud*’ OR ‘follow up stud*’ OR ‘followup stud*’ OR cohort*)
S10	S5 OR S6 OR S7 OR S8 OR S9
S9	SU ((occupational N3 (health OR safety))) OR SU ((work* N3 (health OR safety)))
S8	TI ((occupation* N3 (health OR safety))) OR TI ((work* N3 (health OR safety)))
S7	DE ‘INDUSTRIAL hygiene’
S6	AB ((incentive* OR fund* OR subsid* OR recogni* OR award* OR inspect* OR audit* OR consult* OR sanction* OR penalt* OR fine* OR prosecution* OR ‘enforceable undertaking*’ OR ‘order to comply’ OR citation* OR notification* OR violation* OR breach* OR enforce* OR regulation)) OR AB (((information* OR awareness OR training) N3 (campaign* OR initiative* OR program*)))
S5	TI (incentive* OR fund* OR subsid* OR recogni* OR award* OR inspect* OR audit* OR consult* OR sanction* OR penalt* OR fine* OR prosecution* OR citation* OR notification* OR violation* OR breach* OR ‘enforceable undertaking*’ OR ‘order to comply’ OR information* OR awareness OR training OR enforce* OR regulation)
S4	S1 OR S2 OR S3
S3	(((DE ‘WORK environment’) OR (DE ‘WORK environment ‐‐ Psychological aspects’ OR DE ‘WORK environment ‐‐ Research’)) OR (DE ‘ERGONOMICS’)) OR (DE ‘WORK environment ‐‐ Social aspects’)
S2	AB ((work* OR company OR companies* OR firm OR firms OR organization* OR organisation* OR business* OR institut* OR employe* OR worker* OR staff* OR job) N5 (environ* OR occupational))
S1	TI (work* OR company OR companies* OR firm OR firms OR organization* OR organisation* OR business* OR institut* OR employe* OR worker* OR staff* OR job)) AND TI ((environ* OR occupation*))

#### 3.11.5 Hand search in targeted journals

We will conduct a hand search of the below mentioned journals, to make sure that all relevant articles are found. The hand search will focus on editions published between 2015 and the present to secure recently published articles which have not yet been indexed in the bibliographic databases. This means that the hand search will be date limited to 2015‐present, whereas the rest of the search will not be limited by date.

- *Work & Stress*
- *Policy and Practice in Health and Safety*
- *American Journal of Industrial Medicine*
- *Journal of Labor Economics*
- *Journal of Occupational and Environmental Medicine*
- *International Journal of Environmental Health Research*
- *Scandinavian Journal of Work Environment and Health*
- *Safety Science*
- *Regulation and Governance*
- *Work*
- *Accident Analysis & Prevention*
- *Safety and Health at Work*
- *Journal of Industrial Relations*
- *Law and Policy*
- *Industrial Law Journal*
- *Journal of Safety Research*

Furthermore, we will select additional journals to hand search by identifying the journals with the highest hit‐rate in the electronic searches. The complete list of selected journals for hand searching will be listed in the final report.

#### 3.11.6 Grey literature searches

We will search for such references as dissertations, working papers and conference proceedings, reports, and other EGM's or systematic reviews. Most of the resources searched may include multiple types of references, both published and unpublished. In general, there is a great amount of overlap between the types of references in the chosen resources. The resources are listed once under the category of literature we expect to be most prevalent in the resource, even though multiple types of unpublished/published literature might be identified in the resource. A final list of resources will be included in an appendix to the EGM report.

##### Dissertations

We will search the following resources for dissertations:

- ProQuest Dissertations & Theses Global (ProQuest)
- EBSCO Open Dissertations (EBSCO‐host)

##### Working papers and conference proceedings

We will search the following resources for working papers/conference proceedings:

- Open Grey: http://www.opengrey.eu/
- Google Scholar: https://scholar.google.com/
- Social Science Research Network: https://www.ssrn.com/index.cfm/en/
- OECD iLibrary: https://www.oecd-ilibrary.org/
- NBER working paper series: http://www.nber.org
- Conference Proceedings Citation Index
- Index of Conference Proceedings.

##### Reports

- The International Labour Organisation (ILO): https://www.ilo.org/global/publications/lang--en/index.htm
- World Health Organisation—IRIS: https://apps.who.int/iris/
- The European Agency for Safety and Health at Work (EU‐OSHA): https://osha.europa.eu/en
- European Union Senior Labour Inspectors Committee: https://ec.europa.eu/social/main.jsp?catId=148%26intPageId=685
- European Foundation for the Improvement of Living and Working Conditions (Eurofound): https://www.eurofound.europa.eu/
- United States Department of Labor ‐ Occupational Safety and Health Administration: https://www.osha.gov/
- Canadian Centre for Occupational Health and Safety: https://www.ccohs.ca/
- The Health and Safety Executive (UK): www.hse.gov.uk
- Safe Work Australia: https://www.safeworkaustralia.gov.au/
- WorkSafe (New Zealand): https://www.worksafe.govt.nz/
- The Danish Working Environment Authority (Arbejdstilsynet): https://at.dk
- The Swedish Work Environment Authority (Arbetsmiljöverket): https://www.av.se/
- The Norwegian Labour Inspection Authority (Arbeidstilsynet): https://www.arbeidstilsynet.no/en/
- CORE—research outputs from international repositories: https://core.ac.uk/
- Google searches: https://www.google.com/
- Best Evidence Encyclopedia: http://www.bestevidence.org/
- The Danish National Research Centre for the Working Environment (NFA): https://nfa.dk/
- The National Institute of Occupational Health in Norway (STAMI): https://stami.no/
- Finnish Institute of Occupational Health (FIOH): https://www.ttl.fi/en/
- ANU—The National Research Centre for OHS Regulation (NRCOHSR): http://regnet.anu.edu.au/research/centres/national-research-centre-ohs-regulation-nrcohsr
- Institute for Work & Health: https://iwh.on.ca
- National Institute for Occupational Safety and Health (NIOSH): https://www.cdc.gov/niosh/
- Federal Institute for Occupational Safety and Health (BAuA): https://www.baua.de/EN/Home/Home_node.html

##### Evidence and gap maps and systematic reviews

To locate any potential EGMs or systematic reviews, we will search the following resources:

- 3ie Systematic Review Database: https://www.3ieimpact.org/evidence-hub/publications/systematic-reviews
- 3ie Evidence and Gap Map Repository: https://www.3ieimpact.org/evidence-hub/evidence-gap-maps
- Evidence‐Based Synthesis Program (Department of Veteran Affairs): https://www.hsrd.research.va.gov/publications/esp/
- Evidence based policing matrix: https://cebcp.org/evidence-based-policing/the-matrix/
- Campbell Systematic Reviews Journal: https://onlinelibrary.wiley.com/journal/18911803?af=R
- Cochrane Library: https://www.cochranelibrary.com/
- EPPI‐Centre publications: https://eppi.ioe.ac.uk/cms/Default.aspx?tabid=116
- PROSPERO: https://www.crd.york.ac.uk/prospero/
- Open Science Framework: https://osf.io/
- Epistemonikos: https://www.epistemonikos.org/

##### Trial registries

- AEA Social Science RCT Registry: https://www.socialscienceregistry.org/
- ClinicalTrials.gov: https://clinicaltrials.gov/
- WHO International Clinical Trials Registry Platform: https://www.who.int/clinical-trials-registry-platform
- CENTRAL Trials Register within the Cochrane Library: https://www.cochranelibrary.com/central

We have consulted the list of websites comprised in the article by Kugley et al. (2017).

##### Citation‐tracking and snowballing methods of systematic reviews and EGMs

EGMs or systematic reviews identified during the search process will be citation‐tracked to identify relevant references (further reviews, EGMs, or individual studies). Furthermore, we will utilise forwards citation‐tracking methods on key systematic reviews or EGMs in Google Scholar and Web of Science to identify references that have cited these key reviews or EGMs. The systematic reviews and EGMs identified and selected for citation‐tracking will be listed in the search reporting section of the EGM.

##### Citation‐tracking and snowballing methods of individual studies/references

We will identify the most recently published relevant studies for citation‐tracking. We will select studies from the pool of included references after the title/abstract screening is finished. Since the topic of the EGM covers a number of academic fields, we will not predefine an exact number of individual studies that will be selected for citation‐tracking, but we expect to identify the approximately 20 most cited studies in the field for citation‐tracking. We will select studies based on a citation analysis based on the search terms presented in the search string above, which will be performed in Google Scholar and Web of Science. The studies selected for citation‐tracking/snowballing (including the most recent and most cited) will be listed in the search reporting section of the EGM.

##### Contact to experts

We will contact experts in the field to identify unpublished or on‐going studies found in the searches. We expect primarily to contact authors of reviews/gap maps, as well as authors with many publications on their curriculum and/or authors of newly published works.

### 3.12 Analysis and presentation

#### 3.12.1 Report structure

The EGM report will include the following sections: executive summary, background, methods, results, and conclusion. The executive summary will summarise the report, providing the main findings and their implications for policy and research. The background will provide a description of the burden of working environment hazards to people and societies, state the objectives of the EGM, and define the intervention/outcomes framework. In presenting the methodology used, we will include a thorough description of data sources and methods of searching, the inclusion and exclusion criteria, screening, quality appraisal, data extraction, and the approach to visualisation of the results. This section will provide an example of a full search from a database as well as a PRISMA flow chart. Full search strategies used for each database will be available in an appendix. The results section will present the number, type, and quality of studies retrieved for the intervention and outcome categories. It is likely that the interventions included in this EGM will be complex and multi‐faceted, with single interventions potentially including elements from more than one of the intervention categories included in our conceptual framework. This may, for example, be the case if a study examines the effectiveness of an intervention encompassing elements of both inspection and information/guidance. We will pay particular attention to such instances in which an intervention incorporates elements from more than one conceptual category. Our approach in such cases will be as follows: If an intervention (and the study reporting on it) fits more than one intervention category, it will be listed under each relevant intervention category (meaning that the same intervention may be represented under several intervention categories). This approach is chosen since the primary purpose of the map is to show the existence of evidence for each particular combination of intervention and outcome, and some interventions (and studies) may provide evidence relevant to more than one such combination. To provide clarity and context for users of the map, the EGM report will include background information and contextual details for the interventions included in the map, with clarification of instances in which single interventions incorporate several conceptual intervention categories. Finally, we will present areas of particular interest in depth, for example, areas characterised by a strong evidence base, or conversely, areas in which evidence gaps are evident.

Finally, in the concluding section of the report, we will discuss the implications of the EGM for researchers, decision‐makers and other important stakeholders. We will describe all deviations from the published protocol with reasons.

The report will include the following tables and figures:

- Conceptual model,
- PRISMA flow chart,
- Number of studies by study design,
- Number of studies by intervention and subcategories,
- Number of studies by country/region and population subgroup, if relevant,
- Number of studies by publication type (report, journal article, etc.).

#### 3.12.2 Filters for presentation

Interventions and outcomes will make up the primary dimensions of the map, with coding of the following filters for primary studies and reviews, where appropriate:

1. Study design (RCT, NRS, SR),
2. Country/region and population subgroup, if relevant,
3. Publication type (report, journal article, etc.).

#### 3.12.3 Dependency

In some cases, several studies may have used the same sample of data or some studies may have used only a subset of a sample used in another study. We will include all such studies in the map. If we find multiple reports for a single study, for example, both working papers and journal articles, we will use the latest or most complete version in the map.^3^ If different papers report different analyses, for example on different outcomes or for different subgroups, each paper will be included. It is likely that the systematic reviews we find will include the RCTs in the map, and that more than one systematic review will include the same RCT. All relevant randomised trials will be included, whether they are included in a systematic review or not.

### 3.13 Data collection and analysis

#### 3.13.1 Screening and study selection

Under the supervision of review authors, two review team assistants will first independently screen titles and abstracts to exclude studies that are clearly irrelevant. Studies considered eligible by at least one assistant or studies where there is insufficient information in the title and abstract to judge eligibility will be retrieved in full text. The full texts will then be screened independently by two review team assistants under the supervision of the review authors. Any disagreement of eligibility will be resolved by the review authors. Exclusion of studies that otherwise might be expected to be eligible will be documented and presented in an appendix.

The study inclusion criteria will be piloted by the review authors (see Appendix B: *First and second level screening*). The overall search and screening process will be illustrated in a flow diagram. None of the review authors will be blind to the authors, institutions, or journals responsible for the publication of articles.

### 3.14 Data extraction and management

Two review team assistants will independently extract full bibliographic information along with the information necessary to construct the map (i.e., interventions, outcomes, and filters) using a piloted coding tool (see Appendix). The extraction will be supervised by the review authors. Our coding categories for data extraction will be based on our intervention/outcomes framework. In addition, we will collect details on characteristics that may be of particular interest to decision makers as filters for the evidence.

#### 3.14.1 Tools for assessing risk of bias/study quality of included reviews

Since systematic reviews are often used for decision making, we will appraise the quality of systematic reviews using AMSTAR‐2 (Shea et al., 2017), with two additions to the tool: (1) systematic reviews will be assessed on whether the authors present a plan for handling dependent effect sizes, and (2) systematic reviews will be assessed on whether risk of bias assessments of included studies were performed in duplicate, that is, performed independently by two reviewers after which a consensus coding was agreed upon.

Quality appraisal of systematic reviews will be performed in duplicate, that is, critical appraisal will be performed independently by two reviewers after which a consensus coding will be agreed upon. In case of disagreements that cannot be reconciled between the two reviewers, a third reviewer will make the final assessment.

We will not assess the quality of included primary studies, since the purpose of the EGM is to identify primary effectiveness studies, not to make decisions on single trials (Welch et al., 2019).

#### 3.14.2 Methods for mapping

We plan to use the EPPI‐Mapper software, powered by EPPI‐Reviewer, to generate an online, interactive map. The interventions and outcomes will serve as the primary dimensions of the map. In addition to interventions and outcomes, the following filters will be coded for primary studies (and reviews where appropriate):

- Study designs.
- Country/region and population subgroups, if relevant.
- Publication type (report, journal article, etc.).

## CONTRIBUTIONS OF AUTHORS

**Content**:

*Jan H. Pejtersen, Hermann Burr, Peter Hasle, and Émile Tompa*

*Jan H. Pejtersen* is a senior researcher, Ph.D., and has worked with occupational health and safety for more than 25 years. Jan has studied the association between psychosocial work environment and health both in population studies and in studies at the workplace level. Jan is the author and coauthor of several peer‐reviewed articles in the field of occupational health and safety.

*Hermann Burr*, *Peter Hasle*, and *Émile Tompa* are all researchers with significant expertise and experience with occupational health and safety issues.

**EGM methods**:

*Trine Filges and Anja Bondebjerg*

*Trine Filges* holds a Ph.D. in Economics and has extensive experience as a systematic reviewer and methodologist, having completed a number of systematic reviews in social welfare topic areas as well as in the field of education. Trine has published a number of Campbell Systematic reviews as well as systematic and meta‐analytic reviews in high‐impact journals. Trine's fields of expertise are systematic review methods and statistical analysis.

*Anja Bondebjerg* holds a Master's degree in Sociology and has worked extensively with systematic reviews and research mappings, primarily in the fields of education and early childhood education and care. Anja's field of expertise is systematic review methods.

Both Trine and Anja are currently authoring or co‐authoring a number of other Campbell systematic reviews.

**Information retrieval**:

*Bjørn C. A. Viinholt, Kirsten Birkefoss, and Elizabeth Bengtsen*

*Bjørn C. A. Viinholt* (information specialist) has 4 years of experience in developing and writing systematic reviews. As a part of undertaking systematic reviews, Bjørn has experience in developing systematic search strategies and processes of reference management. Bjørn will contribute to the development of the systematic search strategy and the execution of searches as well as providing assistance with reference management and grey literature searches. Bjørn will also assist with aspects relating to systematic literature searches in Campbell review methodology.

*Kirsten Birkefoss* supports the professional work at VIVE as a search specialist and conducts systematic literature searches for VIVE's many projects and research publications. Kirsten is a consultant at VIVE and has with her experience of many years as a research librarian and search specialist at several research and governmental institutions profound knowledge about database searching in a wide range of subjects, that is, health, labour market, the social field, education, organisation and economics.

*Elizabeth Bengtsen* (information specialist) is an experienced research librarian and search specialist who has worked for core research institutions in Denmark for many years, including The Danish National Research Centre for the Working Environment. With her experience and expertise, Elizabeth will contribute to the EGM both with her information retrieval skills, her profound insight into the research field, and her familiarity with systematic review methodology.

## DECLARATIONS OF INTEREST

None of the authors hold any conflicts of interest in this EGM.

## PLANS FOR UPDATING THE EGM

Anja Bondebjerg and Trine Filges will be responsible for regular updates of the EGM, but this is subject to financing being available.

## SOURCES OF SUPPORT

**Internal sources**

- VIVE, The Danish Center for Social Science Research, Denmark

## Supporting information

Supporting information.

## Footnotes

^1^
We will use AMSTAR‐2 with two additions; see ‘Tools for assessing risk of bias/study quality of included reviews’.

^2^

http://www.prisma-statement.org/PRISMAStatement/. Accessed January 7, 2021.

^3^
That is, if we find several reports of the same study (i.e., with the same outcomes), we will include the version that reports most fully on the outcomes, or, if there is no difference between reports, we will include the latest edition.
