# Supplementary material for: PROTOCOL: Occupational health and safety regulatory interventions to improve the work environment: An evidence and gap map of effectiveness studies
Source: Campbell Syst Rev. 2022 Apr 12;18(2):e1231. doi: 10.1002/cl2.1231 (PMC9005925; doi:10.1002/cl2.1231)
Supplement: Supplementary file 1 — Supporting information. [file CL2-18-e1231-s001.docx]

# Appendices

## 1 Link to online interactive EGM

Add link at the full report stage

## 2 First and second level screening

First level screening is on the basis of titles and abstracts. Second level is on the basis of full texts.

Reference id. no.:                                 Reviewers initials:                                               Source:                                                                         Year of publication:                                                                Country/countries of origin:                                                                            Author(s):                                                                                          The study will be excluded if one or more of the answers to questions 1-3 are ‘No’. If the answers to questions 1 to 3 are ‘Yes’ or ‘Uncertain’, then the full text of the study will be retrieved to assess second level eligibility. All unanswered questions need to be posed again on the basis of the full text. If insufficient information is available, or if the study details are unclear, the authors of the study will be contacted if possible.

**Screening questions:**

*1. Does the study measure the effects of eligible working environment regulatory interventions, as specified in the protocol?* Yes -include

No – stop here and exclude

Uncertain – include

Question 1 guidance: We will only include interventions initiated by working environment regulatory authorities or agencies, thus excluding interventions started by individual businesses or employers at their own initiative. The following categories of intervention will be eligible: formulation of regulatory standards, incentives for compliance, inspection by regulatory agencies, enforcement by regulatory agencies (sanctions), information, guidance, and consulting, and finally training initiatives. Please refer to the protocol for examples. If in doubt, include for second level screening on full text.                                                                                          *2. Does the study measure effects for workers above the age of 15 from workplaces within the OECD?*                         Yes – include

No – stop here and exclude

Uncertain – include                                                                                                                      Question 2 guidance: The population of relevance to this EGM includes workers above the age of 15 and their workplaces. We limit our scope to workplaces located in nations within the OECD. Note here that it is the workplaces that must be located in OECD countries, whereas workers in these workplaces may be citizens of all countries.

*3. Is the report/article a primary quantitative study with a control or comparison condition or a systematic review of effectiveness studies?*                                                                    Yes – include

No – stop here and exclude

Uncertain – include

Question 3 guidance: We are only interested in primary quantitative studies with a control or comparison group or systematic reviews of effectiveness studies. For specification of included designs, see the section entitled “Types of study designs” in the protocol.

If in doubt, include for second level screening on full text. Please include all systematic reviews of topic relevance for second level screening by the review authors.

## 3 Justification of exclusion of studies using an instrumental variable (IV) approach

Studies using instrument variables (IV) for causal inference in non-randomised studies will not be included as the interpretation of IV estimates is challenging. IV only provides an estimate for a specific group namely, people whose behaviour change due to changes in the particular instrument used. It is not informative about effects on never-takers and always-takers because the instrument does not affect their treatment status. The estimated effect is thus applicable only to the subpopulation whose treatment status is affected by the instrument. As a consequence, the effects differ for different IVs and care has to be taken as to whether they provide useful information. The effect is interesting when the instrument it is based on is interesting in the sense that it corresponds to a policy instrument of interest. Further, if those that are affected by the instrument are not affected in the same way the IV estimate is an average of the impacts of changing treatment status in both directions, and cannot be interpreted as a treatment effect. To turn the IV estimate into a LATE requires a monotonicity assumption. The movements induced by the instrument go in one direction only, from no treatment to treatment. The IV estimate, interpreted as a LATE, is only applicable to the complier population, those that are affected by the instrument in the ‘right way’. It is not possible to characterise the complier population as an observation’s subpopulation cannot be determined and defiers do not exist by assumption.

In the binary-treatment–binary-instrument context, the IV estimate can, given monotonicity, be interpreted as a LATE; i.e. the average treatment effect for the subpopulation of compliers. If treatment or instruments are not binary, interpretation becomes more complicated. In the binary-treatment–multivalued-instrument (ordered to take values from 0 to *J*) context, the IV estimate, given monotonicity, is a weighted average of pairwise LATE parameters (comparing subgroup *j* with subgroup *j*−1). The IV estimate can thus be interpreted as the weighted average of average treatment effects in each of the *J* subgroups of compliers. In the multivalued-treatment (ordered to take values from 0 to *T*) – multivalued-instrument (ordered to take values from 0 to *J*) context, the IV estimate for *each pair of instrument values*, given monotonicity, is a weighted average of the effects from going from *t*-1 to *t* for persons induced by the change in the value of the instrument to move from any level below *t* to the level *t* or any level above. Persons can be counted multiple times in forming the weights.

Bibliography:

- Angrist, J.D., & Pischke, J.S. (2009*). Mostly Harmless Econometrics: An Empiricist’s Companion.*Princeton, NJ: Princeton University Press.
- Heckman, J.J. & Urzúa, S. (2010). Comparing IV with structural models: What simple IV can and cannot identify. *Journal of Econometrics, 156*, 27-37.
- Heckman, J.J., Urzúa, S. & Vytlacil, E. (2006). Understanding instrumental variables in models with essential heterogeneity. *The Review of Economics* *and Statistics, 88*(3), 389-432.

## 4 Data extraction

| **Names of author(s)** |
| --- |
| **Title** |
| **Language** |
| **Journal** |
| **Year** |
| **Country** |
| **Programme feature:***Study design* (brief description) |
| **Programme feature:** *Intervention*(intervention type) |
| **Programme feature:***Outcome*(state outcomes used in study) |
| **Programme feature:***Population*(country and subgroup) |
| **Study feature (all studies):***Publication type* |
| **Study feature (systematic reviews):***AMSTAR-rating* |
